# Effects of the CB1 Receptor Antagonists AM6545 and AM4113 on Insulin Resistance in a High-Fructose High-Salt Rat Model of Metabolic Syndrome

**DOI:** 10.3390/medicina56110573

**Published:** 2020-10-29

**Authors:** Basma G. Eid, Thikryat Neamatallah, Abeer Hanafy, Hany M. El-Bassossy, Hibah M. Aldawsari, Kiran Vemuri, Alexandros Makriyannis

**Affiliations:** 1 Department of Pharmacology and Toxicology, Faculty of Pharmacy, King Abdulaziz University, Jeddah 21589, Saudi Arabia; taneamatallah@kau.edu.sa (T.N.); ahanafyvet@yahoo.com (A.H.); 2 Department of Pharmacology, Faculty of Veterinary Medicine, Kafrelsheikh University, Kafrelsheikh 33516, Egypt; 3 Department of Pharmacology and Toxicology, Faculty of Pharmacy, Zagazig University, Zagazig 44519, Egypt; helbassossy@pharmacy.zu.edu.eg; 4 Department of Pharmaceutics, Faculty of Pharmacy, King Abdulaziz University, Jeddah 21589, Saudi Arabia; haldosari@kau.edu.sa; 5 Center for Drug Discovery, Northeastern University, Boston, MA 02115, USA; kiranvvemuri@gmail.com (K.V.); a.makriyannis@northeastern.edu (A.M.); 6 Departments of Chemistry and Chemical Biology and Pharmaceutical Sciences, Northeastern University, Boston, MA 02115, USA

**Keywords:** metabolic syndrome, cannabinoids, AM6545, AM4113, insulin resistance

## Abstract

*Background and Objectives:* Insulin resistance (IR) is a serious condition leading to development of diabetes and cardiovascular complications. Hyper-activation of cannabinoid receptors-1 (CB1) has been linked to the development of metabolic disorders such as IR. Therefore, the effect of blocking CB1 on the development of IR was investigated in the present study. *Materials and Methods:* A 12-week high-fructose/high-salt feeding model of metabolic syndrome was used to induce IR in male Wistar rats. For this purpose, two different CB1-antagonists were synthesized and administered to the rats during the final four weeks of the study, AM6545, the peripheral neutral antagonist and AM4113, the central neutral antagonist. *Results:* High-fructose/salt feeding for 12 weeks led to development of IR while both AM6545 and AM4113, administered in the last 4 weeks, significantly inhibited IR. This was correlated with increased animal body weight wherein both AM6545 and AM4113 decreased body weight in IR animals but with loss of IR/body weight correlation. While IR animals showed significant elevations in serum cholesterol and triglycerides with no direct correlation with IR, both AM6545 and AM4113 inhibited these elevations, with direct IR/cholesterol correlation in case of AM6545. IR animals had elevated serum uric acid, which was reduced by both AM6545 and AM4113. In addition, IR animals had decreased adiponectin levels and elevated liver TNFα content with strong IR/adiponectin and IR/TNFα correlations. AM6545 inhibited the decreased adiponectin and the increased TNFα levels and retained the strong IR/adiponectin correlation. However, AM4113 inhibited the decreased adiponectin and the increased TNFα levels, but with loss of IR/adiponectin and IR/TNFα correlations. *Conclusions:* Both CB1 neutral antagonists alleviated IR peripherally, and exerted similar effects on rats with metabolic syndrome. They also displayed anti-dyslipidemic, anti-hyperurecemic and anti-inflammatory effects. Overall, these results should assist in the development of CB1 neutral antagonists with improved safety profiles for managing metabolic disorders.

## 1. Introduction

Insulin resistance is a key component of the metabolic syndrome in addition to obesity and dyslipidemia [1]. The rate of global increase of the cases of metabolic syndrome is alarming and has been attributed partially to dietary imbalances and lifestyle. Metabolic syndrome has a global prevalence of 10–84% with a variety of variables such as race, age, ethnicity, sex, environment and socioeconomic factors [2,3]. An overly active endocannabinoid system is a major culprit in the development of obesity and its metabolic complications [4]. Insulin resistance, lipogenesis and appetite regulation are also regulated by the endocannabinoid system [5].

Hyper-activation of cannabinoid receptor-1 (CB1) by the endogenous ligands anandamide and 2-arachidonoylglycerol (2-AG) was shown to mediate metabolic abnormalities such as insulin resistance and altered homeostasis of lipids [6], while CB1 antagonists have been implicated in treating metabolic disorders as well as obesity [7]. Over the past years, many clinical trials for treating these conditions have been undertaken using rimonabant, a CB1 inverse agonist [8]. In the United States, however, its approval was halted due to the development of unwanted neuropsychiatric side effects [9]. Since then, there has been an urgent need to pursue alternative approaches to modulate CB1 receptor transmission for therapeutic gain. More recently, ongoing efforts have aimed at the development of compounds with improved pharmacological and safety profiles with limited brain permeability. To circumvent the issue of the psychiatric side effects of CB1 inverse agonism, our laboratory undertook a program to develop CB1 receptor blockers that behaved as neutral antagonists, i.e., bound the CB1 receptor with high affinity but without altering secondary signaling protein levels, i.e., cAMP. We hypothesized that CB1 neutral antagonists will be as efficacious as rimonabant in multiple therapeutic endpoints but with fewer or none of the side effect profiles.

Key and subtle manipulations of molecular features have assisted in designing ligands that exhibited varying pharmacological properties. These include no or low inverse agonist activity, along with varying or a minimal tendency to partition into the central nervous system (CNS). Studies have shown that modifying the rimonabant pyrazole CB1 pharmacophore could generate novel CB1 modulators [10]. A peripherally acting biarylpyrazole CB1 antagonist, namely, AM6545, developed in our laboratory, was shown to be a safer alternative to rimonabant for treating metabolic disorders [11,12,13]. AM6545, in vivo, has proven to be efficacious in animal models. AM6545 was largely restricted to the periphery, and unlike rimonabant, it behaved as a neutral antagonist when tested in CB1 cell lines and primary cell cultures [14]. Additionally, we have included in our present study, AM4113, a brain penetrant CB1 neutral antagonist, also developed in our laboratory. Unlike AM6545, which only acts peripherally, AM4113 can act both peripherally and centrally by penetrating the brain. Notably, AM4113 is more potent than rimonabant and is a selective CB1 antagonist that lacks the central side effects associated with rimonabant [15].

It is generally believed that the vast majority of metabolic syndrome effects are initiated through peripheral actions. CB1 receptor antagonists were previously demonstrated to reduce body weight [5] and ameliorate the cardio-metabolic risk profile in obese rodents [16] and humans [17]. The objective of the present study was to examine the effects of the CB1 antagonists AM6545 and AM4113 in a high-fructose/high-salt model of metabolic syndrome in rats. The effects of these drugs have not been previously investigated using this animal model of metabolic syndrome. The goal was to determine their ability to reverse the unhealthy effects associated with the metabolic syndrome such as insulin resistance, as well as to provide evidence indicating that these effects can be modulated by inhibiting CB1 receptors. We hypothesized that AM6545 and AM4113 would potentially prevent the development of insulin resistance component of metabolic syndrome. The role of these CB1 antagonists in correcting the hyperlipidemia, hyperuricemia and low-grade inflammation reported in metabolic syndrome has also been investigated.

## 2. Materials and Methods

### 2.1. AM6545 and AM4113

The CB1 receptor neutral antagonists AM6545 (5-(4-[4-cyanobut-1-ynyl]phenyl)-1-(2,4-dichlorophenyl)-4-methyl-N-(1,1-dioxo-thiomorpholino)-1H-pyrazole-3-carboxamide) and AM4113 (5-(4-alkylphenyl)-1-(2,4-dichlorophenyl)-4-methyl-*N*-(piperidin-1-yl)-1*H*-pyrazole-3-carboxamide) were synthesized at the Center of Drug Discovery, Northeastern University (Boston, MA, USA) [14,18].

### 2.2. Animals

Male Wistar rats of 6 weeks old (weighing 150–190 g) from King Abdulaziz University were housed at room temperature with 12:12 h light/dark cycle and free access to a standard rodent diet and water (4 rats per cage). All experimental protocols were approved by the Unit of Biomedical Ethics Research Committee at King Abdulaziz University (Reference No. 479-16).

### 2.3. Study Protocol

Rats were left one week prior to the experiment for habituation and were fed the standard commercial rodent food with water freely accessed. Rats were then divided into 6 groups (8 rats/group) in a random manner: control group (C) received standard rodents food with free access to drinking water for 12 weeks, metabolic syndrome group (M) received standard rodent food with free access to 20% *w/v* fructose solution + 3% salt for the same above mentioned period. AM6545-treated control group (C+A65) received standard food and water for 12 weeks as well as AM6545 (10 mg/kg/day) intraperitoneally (IP) daily during the last 4 weeks. AM4113-treated control group (C+A41) received standard food and water for 12 weeks as well as AM4113 (10 mg/kg/day) IP daily during the last 4 weeks. AM6545-treated metabolic syndrome (M+A65) and AM4113-treated metabolic syndrome group (M+A41) received standard rodent food with free access to 20% *w/v* fructose solution + 3% salt throughout the entire 12 week period and AM6545 (10 mg/kg/day) IP or AM4113 (10 mg/kg/day) IP, respectively, daily during the last 4 weeks. AM6545 (10 mg/kg/day) and AM4113 (10 mg/kg/day) were suspended in 0.5% carboxymethyl cellulose (CMC). Control and metabolic syndrome groups only received CMC intraperitoneally as a vehicle.

### 2.4. Specimen Collection

Body weights of the animals were recorded at the beginning of the study and at 3, 6, 9 and 12 weeks. At the end of the protocol, the rats were anaesthetized with urethane (1 g/kg) given intraperitoneally. Blood was centrifuged for 20 min at 4000× *g*, 4 °C after collection from the retro-orbital plexus. The serum was aliquoted and kept at −20 °C until analysis for insulin and adiponectin. The liver was rapidly isolated and immediately kept at −80 °C until homogenization for Enzyme-linked immunosorbent assay (ELISA) analysis of TNFα.

### 2.5. Glucose and Oral Glucose Tolerance Test (OGTT)

Blood glucose levels were measured using a glucometer from blood obtained from the tail vein. An oral glucose tolerance test was also performed after an oral load (2 g/kg) of a D-glucose solution (20% *w*/*v*) following an overnight fasting period. Glucose levels were measured at 0, 30, 60, 90, and 120 min directly in blood sampled from the tail vein. Glycemic responses are expressed as the respective area under the OGTT curve (AUC) during the two-hour observation time.

### 2.6. Tissue Homogenates

About 0.5 g of kidney or liver tissue was homogenized in 0.5 mL (phosphate buffered saline + 1% Triton × 100, pH 7.4) using homogenizer (Ortoalresa, Madrid, Spain). This was followed by centrifugation at 4000× *g* for 20 min at 4 °C. The supernatant was collected in Eppendorf tubes and placed in a deep freezer (at −80 °C) for further biochemical experiments.

### 2.7. Biochemical Measurements

An ELISA kit with wells pre-coated with monoclonal anti-rat insulin antibodies (Millipore, Billerica, MA, USA) plate was utilized for serum insulin determination. Insulin resistance index (IR index) was calculated using blood glucose and insulin levels according to the following equation [19]: IR index = glucose (mmol.L^−1^) × insulin (mU.L^−1^)/22.5. The adiponectin level in the serum was determined by a rat adiponectin ELISA Kit (ab108784). The concentration of TNF-α in the liver samples was assayed by an ELISA kit (R&D systems RTA00). An automatic ELISA reader was used to quantify ELISA reactions based on optical density. Serum triglycerides, cholesterol and uric acid were measured by an ELITech assay kit (ELITech; Laindon, Essex, France). Serum levels of the endogenous cannabinoids anandamide (AEA) and 2-arachidonoylglycerol (2-AG) were measured using kits purchased from Real-Gene Labs (Lakeforest, CA, USA).

### 2.8. Statistical Analysis

Results are presented as the mean ± standard error of mean and as box plots showing the data distribution. Statistical analysis was performed by “Kolmogorov–Smirnov” normality test followed by analysis of variance (ANOVA) and Tukey’s post-hoc test using GraphPad Prism (Version 8). Results were significantly different if *p* < 0.05. A parametric correlation analysis was performed, and the correlation coefficient (r) and its *p* value were calculated using the same software, to find the correlation between IR results and various parameters.

## 3. Results

In order to confirm the raised endocannabinoid tone in our model, the liver content of the endogenous cannabinoids anandamide and 2-arachidonoylglycerol were assessed. Table 1 reveals that the metabolic syndrome group (M) had a significantly higher content of both anandamide and 2-AG in comparison to the control group (C). Treatment with AM6545 or AM4113 did not change the anandamide and 2-AG content.

**Figure 1:**
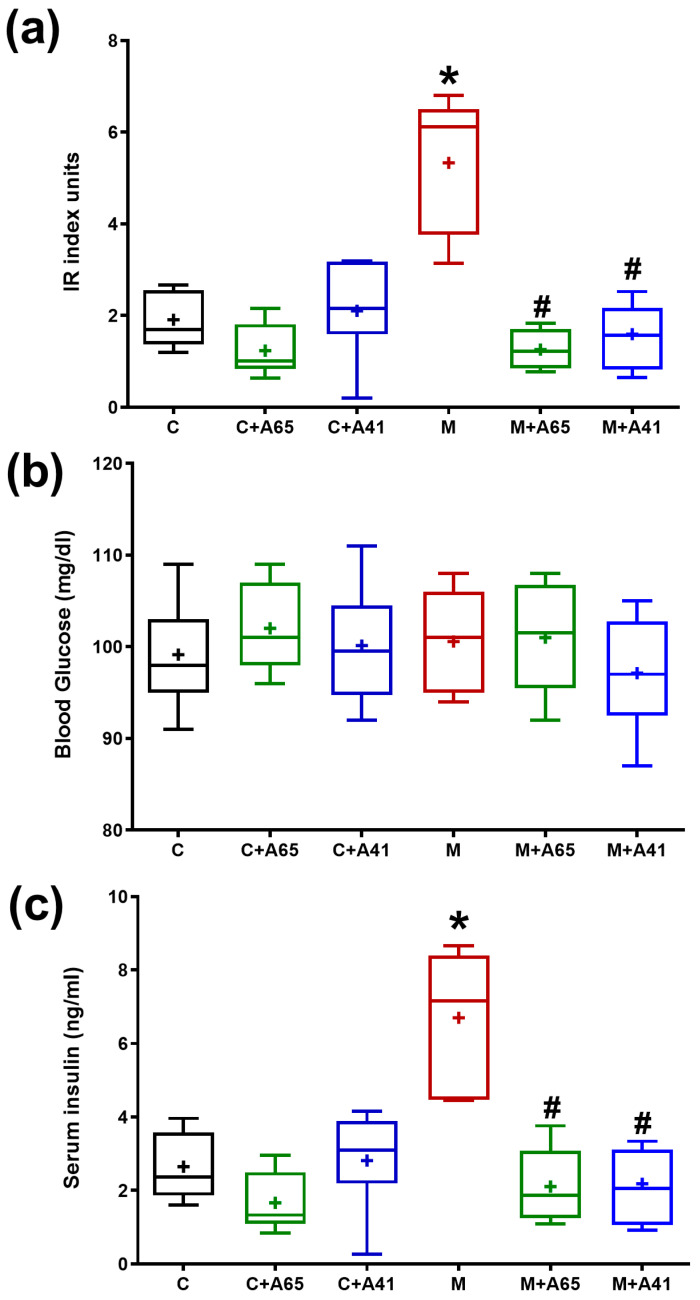
Effect of AM6545 (A65) and AM4113 (A41) on insulin resistance (IR) index (**a**), blood glucose level (**b**) and serum insulin level (**c**) in normal (C) and metabolic syndrome rats (M). Data are presented as box plots with the mean of 8 rats shown as (+). * Significant difference compared with the control group (C) (*p* < 0.05). # Significant difference compared with the metabolic syndrome group (M) (*p* < 0.05) by ANOVA followed by Tukey’s post-hoc test.

Calculation of IR index showed that it was significantly elevated in metabolic syndrome rats relative to the control group (Figure 1a). Treatment of metabolic syndrome rats with either AM6545 (group M + A65) or AM4113 (M + A41) caused a significant decrease in the IR index in comparison with non-treated metabolic syndrome rats, bringing the IR index down to levels comparable with control levels. Blood glucose levels were similar in all groups (Figure 1b). Serum insulin levels were significantly increased in group M relative to the control group (Figure 1c). This was corrected with treatment with either AM6545 (group M + A65) or AM4113 (M + A41), which caused a significant reduction in the insulin levels when compared to the metabolic syndrome group M. Treatment of control rats with AM6545 (group C + A65) and AM4113 (C + A41) showed no significant changes (*p* < 0.05) in serum insulin levels relative to group C (Figure 1c).

**Figure 2:**
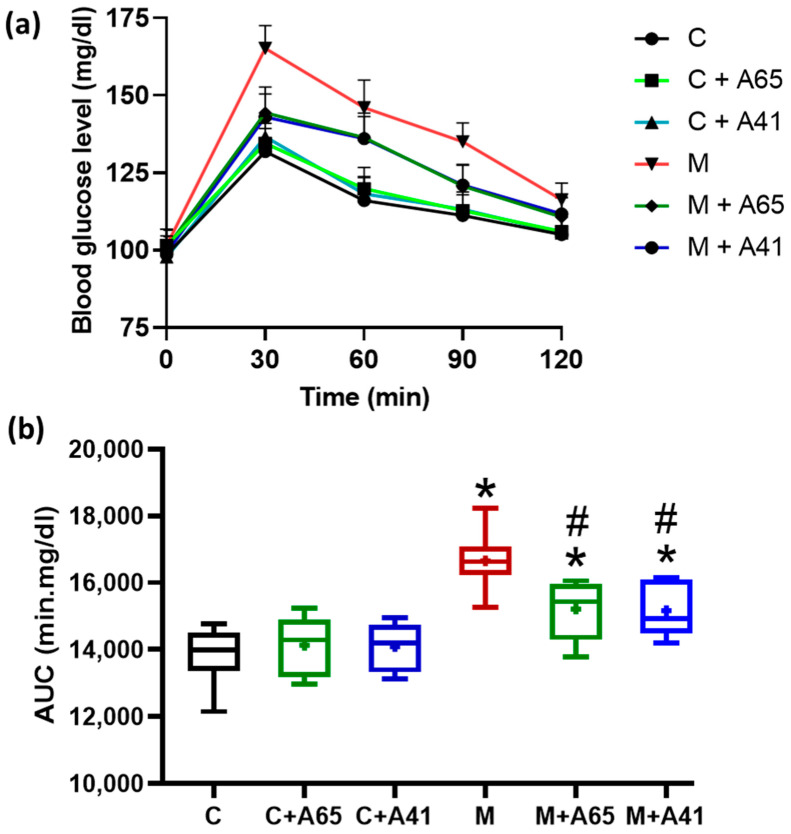
Effect of AM6545 (A65) and AM4113 (A41) on oral glucose tolerance test (OGTT) (**a**), and the respective AUC over the 120 min study time (**b**). Data in (**a**) are shown as the mean ± standard error of 8 rats. Data in (**b**) are presented as box plots with the mean of 8 rats shown as (+). * Significant difference compared with the control group (C) (*p* < 0.05). # Significant difference compared with the metabolic syndrome group (M) (*p* < 0.05) by ANOVA followed by Tukey’s post-hoc test.

Blood glucose levels following an oral glucose tolerance test are shown in Figure 2a, and the corresponding area under the curve AUC is shown in Figure 2b. After an oral challenge with glucose, blood glucose levels reached their peak levels at 30 min and then started to decline in all the groups. It is clear from Figure 2b that the metabolic group exhibited a significantly higher AUC in comparison to the control group C. Treatment with AM6545 and AM4113 resulted in significantly lower AUC than the metabolic syndrome animals.

**Figure 3:**
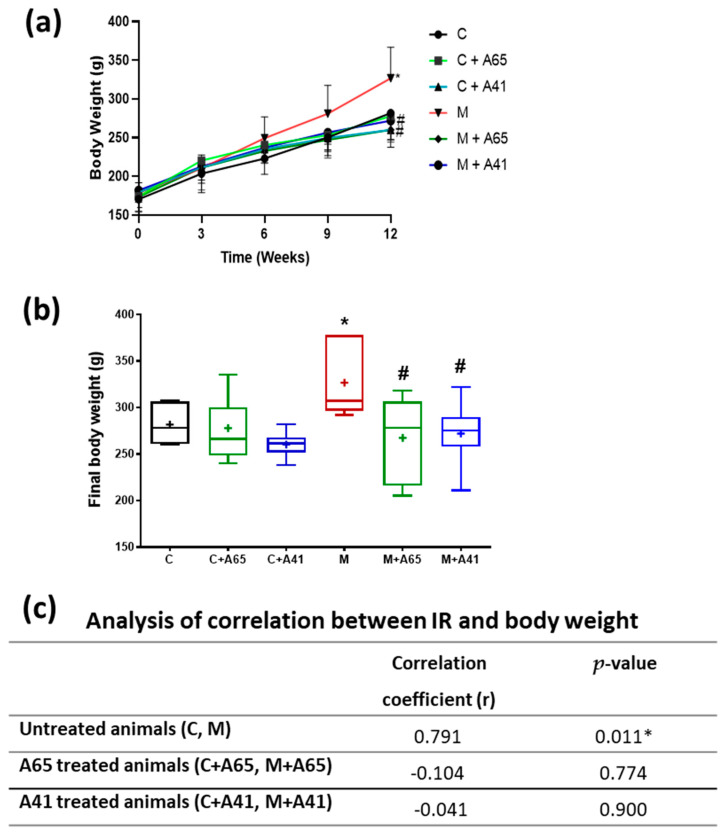
(**a**) Changes in body weight caused by AM6545 (A65) and AM4113 (A41) in normal (C) and metabolic syndrome rats (M) over the 12 weeks of the study shown as the mean ± standard error of 8 rats. (**b**) Final body weights attained in the different experimental groups presented as box plots with the mean of 8 rats shown as (+). (**c**) Effects of AM6545 and AM4113 on the correlation between body weight and IR. * Significant difference compared with the control group (C) (*p* < 0.05). # Significant difference compared with the metabolic syndrome group (M) (*p* < 0.05) by ANOVA followed by Tukey’s post-hoc test.

Figure 3a shows the changes in body weight over the time-course of the study. All groups showed a steady increase in body weight over 12 weeks. Measurements of final body weight, revealed that twelve weeks of high-fructose/high-salt water ingestion (Group M) resulted in a significant rise in body weight, in comparison to the control group (Group C), as indicated in Figure 3b. Treatment with AM6545 and AM4113 (Group M+A65 and Group M + A41, respectively) reversed the increase in the body weight seen in the metabolic group and retained the body weights of these animals similar to control levels (Figure 3b). Figure 3c shows that there is a strong correlation between IR and body weight. Although treatment with AM6545 and AM4113 prevented the increased body weight, the correlation between IR and body weight was not present after treatment with AM6545 or AM4113.

**Figure 4:**
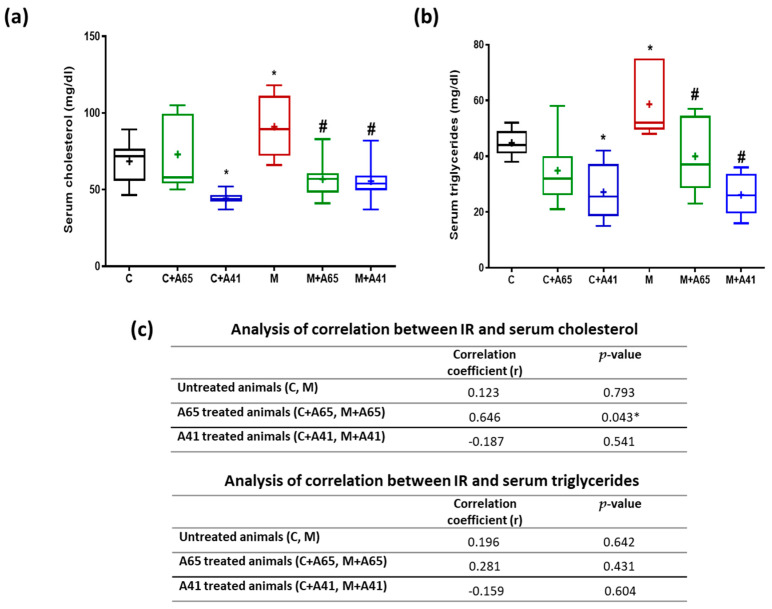
Effect AM6545 (A65) and AM4113 (A41) on serum cholesterol level (**a**) and serum triglycerides (**b**) in normal (C) and metabolic syndrome rats (M). (**c**) Effects of AM6545 and AM4113 on the correlation between IR and serum cholesterol, and IR and serum triglycerides. Data in (**a**) and (**b**) are presented as box plots with the mean of 8 rats shown as (+). * Significant difference compared with the control group (C) (*p* < 0.05). # Significant difference compared with the metabolic syndrome group (M) (*p* < 0.05) by ANOVA followed by Tukey’s post-hoc test.

Figure 4a shows a significant increase in cholesterol level in group M compared with group C. Treatment with of metabolic rats with AM6545 (group M + A65) and AM4113 (M + A41) resulted in a significant reduction in cholesterol levels when compared to the metabolic syndrome group (M). Meanwhile, treatment of control rats with AM6545 (group C + A65) showed no significant changes (*p* < 0.05) in cholesterol levels relative to group C (Figure 4a). On the other hand, treatment of control rats with AM4113 (C + A41) caused a significant reduction in cholesterol levels relative to the control group C. There was a significant increase in triglycerides (TGs) level in the metabolic syndrome rats relative to control rats (Figure 4b). Metabolic syndrome rats treated with AM6545 (group M + A65) and AM4113 (M + A41) showed decreased TGs level compared with the metabolic syndrome rats (M). On the other hand, control rats treated with AM6545 (group C + A65) showed no significant change in TGs level relative to the control group C (Figure 4b). However, when given to control rats AM4113 (C + A41) significantly reduced their triglyceride levels (Figure 4b). Interestingly, the correlation between the elevated serum cholesterol/triglycerides and IR induction was not significant. While both AM6545 and AM4113 inhibited these elevations, AM6545 had a direct IR/cholesterol correlation (Figure 4c).

**Figure 5:**
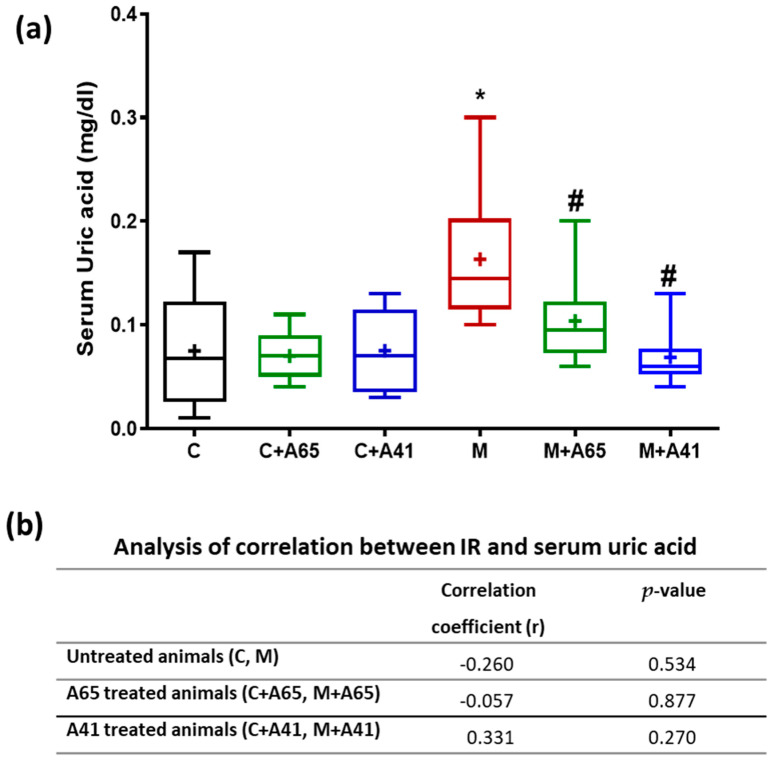
(**a**) Effects of AM6545 (A65) and AM4113 (A41) on serum uric acid levels in normal (C) and metabolic syndrome rats (M). (**b**) Effects of AM6545 and AM4113 on the correlation between IR and serum uric acid levels. Data are presented in (**a**) as box plots with the mean of 8 rats shown as (+). * Significant difference compared with the control group (C) (*p* < 0.05). # Significant difference compared with the metabolic syndrome group (M) (*p* < 0.05) by ANOVA followed by Tukey’s post-hoc test.

Furthermore, serum uric acid levels were measured and compared in the different animal groups (Figure 5). The level of uric acid was much greater in the metabolic syndrome animals (group M) in comparison to the controls (group C) (Figure 5a). This level was significantly lower after treatment of the metabolic syndrome animals with AM6545 (group M + A65) or AM4113 (M + A41). Control rats treated with AM6545 (group C + A65) and AM4113 (C + A41) showed no significant change in uric acid levels relative to the control group C (Figure 5a). An analysis of correlation between IR and uric acid levels revealed that there was no direct correlation between these two parameters in all groups (Figure 5b).

**Figure 6:**
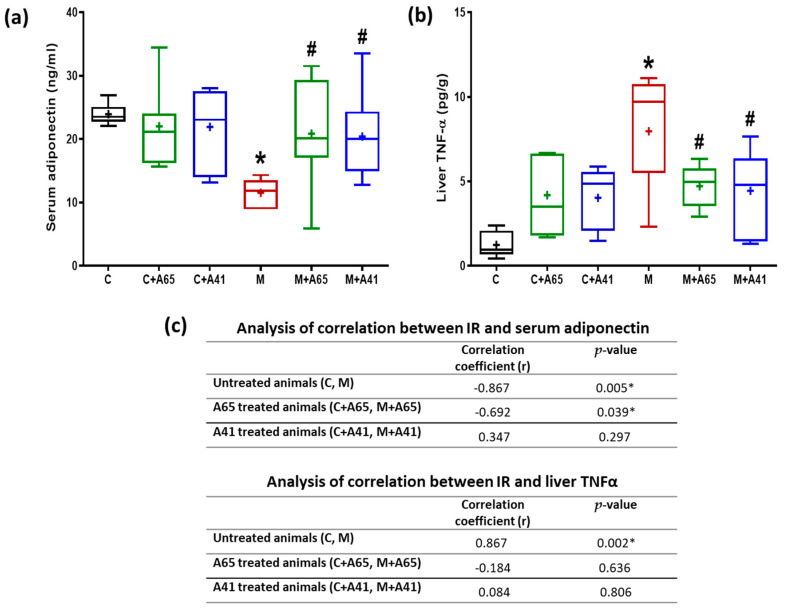
Effect of AM6545 (A65) and AM4113 (A41) on serum adiponectin levels (**a**) and liver content of TNF-α (**b**) in normal (C) and metabolic syndrome rats (M). (**c**) Effects of AM6545 and AM4113 on the correlation between IR and serum adiponectin, and between IR and liver TNF-α. Data are presented in (**a**) and (**b**) as box plots with the mean of 8 rats shown as (+). * Significant difference compared with the control group (C) (*p* < 0.05). # Significant difference compared with the metabolic syndrome group (M) (*p* < 0.05) by ANOVA followed by Tukey’s post-hoc test.

Group M serum adiponectin was significantly lower than group C (Figure 6a). This decrease was corrected significantly upon treatment of metabolic syndrome rats with AM6545 (group M + A65) or AM4113 (M + A41), whereas, no significant difference in serum adiponectin level between C + A65, C + A41 and group C was noted (Figure 6a). Figure 6b shows the liver TNF-α levels in the different groups. It is evident that the metabolic group (M) had a significantly higher level of TNF-α, in comparison to the controls (C). Treatment of the metabolic rats with AM6545 (group M + A65) or AM4113 (M + A41) caused TNF-α to decrease and restored it to levels similar to the control group. AM6545 inhibited the decreased adiponectin and the increased TNFα levels with a strong correlation between IR and adiponectin (Figure 6c). However, AM4113 inhibited the decreased adiponectin and the increased TNFα without IR/adiponectin and IR/TNFα correlations.

## 4. Discussion

The role of CB1 receptors in metabolic dysregulation has been well documented wherein activation of CB1 results in increased appetite, increased hepatic lipogenesis and insulin resistance [20]. CB1 is abundantly found in the CNS in areas regulating emotions and feeding patterns and is also peripherally expressed in the liver, intestine and adipose tissue [21]. Previous studies have shown that blocking CB1 receptors by inverse agonists such as rimonabant [7] or CB1–/– mice [6] displayed resistance to diet-induced obesity.

The roles of the peripheral CB1 antagonist AM6545 and the CB1 neutral antagonist AM4113 on insulin resistance development using the high-fructose/high-salt model of metabolic syndrome were investigated in the present study. The metabolic syndrome animals in this study exhibited a hyperactive endocannabinoid tone as indicated by raised hepatic concentrations of the endogenous cannabinoids anandamide and 2-AG (Table 1). It is clear that both CB1 antagonists acted peripherally and blocked different metabolic syndrome components, namely, weight gain, dyslipidemia, hyperinsulinemia and inflammation.

In the present study, high-fructose/high-salt intake caused a state of metabolic syndrome as evidenced by hyperinsulinemia, weight gain, dyslipidemia and hyperuricemia as was previously reported [22]. There was also a strong correlation between IR and body weight gain, while both AM6545 and AM4113 decreased body weight in IR animals but with lost IR/body weight correlation. Furthermore, blocking CB1 peripherally by AM6545 or centrally by AM4113 ameliorated the actions induced by high-fructose/high-salt feeding to near control values as follows: Both AM6545 and AM4113 reduced insulin level and IR index to near control values suggesting that both halted insulin resistance due to high-fructose/high-salt feeding. AM6545 corrects diet-induced IR by blocking liver CB1 receptors [5]. To further substantiate these findings, an OGTT was performed with calculation of the AUC of the OGTT over the 120 min study time. Results showed that metabolic syndrome animals had significantly elevated glycemia in comparison to controls, which was then reduced in the metabolic syndrome groups treated with either AM6545 (M + A65) or AM4113 (M + A41). These data are consistent with our IR findings and further confirm the role of AM6545 and AM4113 in controlling metabolic syndrome-induced hyperglycemia.

The effects of AM6545 and AM4113 on food intake have been previously reported by members of our research group in numerous studies [18,23,24,25]. In the current study, both AM6545 and AM4113 reduced the body weight gain of rats. This is in line with previously published data, which showed that AM4113 suppressed feeding through decreasing appetite [18] and reduced weight gain in rats [26]. In the literature, AM6545 inhibited the intake of food in the short-term, and resulted in lower weights in rats [14] and mice [14].

Hyperlipidemia is a major hallmark of metabolic syndrome. It is characterized by an elevated level of serum lipids due to lipolysis inhibition in the adipose tissue secondary to insulin resistance [27]. This results in free fatty acids that ultimately increase hepatic triglycerides and cholesterol esters. This agrees with previous reports showing elevations in cholesterol and triglycerides. Both AM6545 and AM4113 effectively reduced serum triglycerides and cholesterol levels in the metabolic syndrome group. This is congruent with earlier data showing that AM6545 treatment was previously shown to ameliorate dyslipidemia and insulin resistance in mice by acting on the liver [5,28].

Adiponectin is a serum hormone, derived from adipocytes [29]. Levels of adiponectin are lowered in conditions such as diabetes mellitus [30], hypertension [31], coronary diseases [32] and in obese patients [33], since it is negatively related to the amount of visceral adipose tissue in agreement with the above [34]. Our study shows lowered adiponectin levels in animals suffering from metabolic syndrome. Treatment with AM6545 or AM4113 restored the levels of adiponectin in metabolic syndrome rats which suggests that these compounds may lead to an anti-diabetic, anti-inflammatory and anti-atherogenic effect as is evidenced by the increased adiponectin levels.

On the other hand, fructose feeding resulted in an elevation in TNFα as was previously shown in animal models of obesity [28]. This was reversed by both CB1 blockers confirming their anti-inflammatory action. The occurrence of metabolic syndrome and diabetes type 2 has been linked to low-grade inflammation due to ongoing cytokine release [35]. We have reported increased concentrations of the inflammatory cytokine TNF-α in animal models of metabolic syndrome. In the current study, metabolic syndrome animals showed three-fold rise in TNF-α serum levels while both AM6545 and AM4113 reduced TNF-α to similar levels. This effect is reflected in the observed changes using this animal model of metabolic syndrome.

A key component of metabolic syndrome that may lead to cardiovascular complications is hyperuricemia [36,37,38,39]. It has been reported that rats exposed to high-fructose exhibit insulin resistance as well as hyperuricemia [39]. In the current study, we have demonstrated increased uric acid levels in the serum in metabolic syndrome induced by high-fructose diet. These were significantly lowered after treatment of the metabolic syndrome rats with either AM6545 or AM4113. Therefore, these drugs may be useful in treating the hyperuricemia associated with metabolic syndrome.

## 5. Conclusions

Overall, our results indicate that the neutral CB1 antagonists AM6545 and AM4113 ameliorated IR by acting peripherally. Both exert similar effects on rats that have developed the metabolic syndrome. This supports our conclusion and suggests a common mechanism of action for both compounds, including improvement of insulin resistance, an anti-hyperlipidemic effect and anti-inflammatory action without affecting the blood glucose level. These findings should aid in the developing a novel class of effective drugs for treating obesity and metabolic disorders, without the undesirable psychiatric side effects typically associated with inverse agonists.

## Figures and Tables

**Table 1:** Liver content of anandamide and 2-arachidonoylglycerol (2-AG).

	Control (C)	C + A65	C + A41	M	M + A65	M + A41
Anandamide (pmol/g)	4.34 ± 0.2	4.26 ± 0.35	4.38 ± 0.26	6.81 * ± 0.13	6.63 * ± 0.15	6.58 * ± 0.17
2-AG (nmol/g)	6.465 ± 0.82	6.96 ± 0.83	6.71 ± 0.76	18.54 * ± 1.54	17.03 * ± 1.13	16.28 * ± 1.02

The table shows the anandamide and 2-AG content in the livers of control (C), AM6545-treated control (C+A65), AM4113-treated control (C+A41), metabolic (M), AM6545-treated metabolic (M+A65) and AM4113-treated metabolic (M+A41) rats. Data are presented as mean ± standard error, *n* = 8. * Significantly different from control at *p* < 0.05.
